# Finding full texts in bulk: a comparison of EndNote 20 versus Zotero 6 using the University of York's subscriptions

**DOI:** 10.5195/jmla.2024.1880

**Published:** 2024-07-01

**Authors:** Helen A. Fulbright, Connor Evans

**Affiliations:** 1 helen.fulbright@york.ac.uk Information Specialist/Research Fellow in Information Science Centre for Reviews and Dissemination, University of York, York, United Kingdom; 2 connor.evans@york.ac.uk Research Assistant, Centre for Reviews and Dissemination, University of York, York, United Kingdom

**Keywords:** Full text retrieval, find full texts, find available PDF, endnote, zotero

## Abstract

**Objective:**

To understand the performance of EndNote 20 and Zotero 6's full text retrieval features.

**Methods:**

Using the University of York's subscriptions, we tested and compared EndNote and Zotero's full text retrieval. 1,000 records from four evidence synthesis projects were tested for the number of: full texts retrieved; available full texts retrieved; unique full texts (found by one program only); and differences in versions of full texts for the same record. We also tested the time taken and accuracy of retrieved full texts. One dataset was tested multiple times to confirm if the number of full texts retrieved was consistent. We also investigated the available full texts missed by EndNote or Zotero by: reference type; whether full texts were available open access or via subscription; and the content provider.

**Results:**

EndNote retrieved 47% of available full texts versus 52% by Zotero. Zotero was faster by 2 minutes 15 seconds. Each program found unique full texts. There were differences in full text versions retrieved between programs. For both programs, 99% of the retrieved full texts were accurate. Zotero was less consistent in the number of full texts it retrieved.

**Conclusion:**

EndNote and Zotero do not find all available full texts. Users should not assume full texts are correct; are the version of record; or that records without full texts cannot be retrieved manually. Repeating the full text retrieval process multiple times could yield additional full texts. Users with access to EndNote and Zotero could use both for full text retrieval.

## INTRODUCTION

In evidence synthesis projects, after the initial stage of screening on titles and abstracts, researchers require access to the full texts. Citation management software such as EndNote and Zotero both have options to find full texts in bulk and automatically attach these to the relevant records. However, EndNote and Zotero do not retrieve all full texts, even when these are available open access or through an institution's subscriptions [1]. This results in having to manually search for and download remaining full texts, which can be time-consuming.

Both EndNote and Zotero are widely used by information specialists and researchers for managing records from database searches or other sources. EndNote desktop requires the purchase of a license (which includes software updates but not later releases of the software unless the license is upgraded) [2]. In comparison, Zotero can be used for free with no limits on storage space but no cloud storage. For users who require their data to be synced with Zotero's cloud storage (to work collaboratively, or across multiple devices), the program can be used for free with a limit of up to 300 MB data; it also has subscription tiers which determine the storage space per user or institution [3].

Researchers can use either EndNote or Zotero for screening full texts, although many prefer dedicated systematic review software such as EPPI-Reviewer, Covidence, or Rayyan (though not all systematic review software can interface with EndNote or Zotero). Several institutions have compared the programs’ features alongside other reference management software [4, 5, 6]. However, we are unaware of any evaluations that compare the performance of full text retrieval. This information could help information specialists, researchers, and institutions to make an informed decision about using either program (or both), and whether to purchase or subscribe to them.

This paper aims to inform users about finding full texts using EndNote or Zotero. Its objectives are to understand how each program looks for full texts; test and compare the full text retrieval and accuracy of each program; report on unique full texts (found by one program only); investigate whether document versions vary (where both programs found a full text for the same record); report on the consistency of the number of full texts found using the same dataset multiple times; and explore the common features of full texts missed by EndNote or Zotero.

## METHODS

This study is based on programs available to the authors: EndNote 20 (version 20.4.1) and Zotero 6 (version 6.0.27) [7, 8]. It was conducted due to the Centre for Reviews and Dissemination's (CRD, University of York) need to understand the performance of EndNote versus Zotero. CRD researchers use a variety of programs for screening, including EPPI-Reviewer. As Zotero is now able to interface with this program to bulk import full texts and attach these to the record [9], we wanted to test its performance against EndNote, which we typically use for full text retrieval.

An overview of the methodology is as follows:

1. Communication with EndNote's technical support team and the Zotero Forum to ask questions on their find full text features.
2. Tests of EndNote and Zotero to determine:
  - the number of full texts retrieved;
  - number of available full texts retrieved;
  - unique full texts retrieved (found by one program only);
  - differences in versions of full texts retrieved (where both programs found full texts for the same record);
  - time taken to retrieve the full texts;
  - consistency of the number of full texts found using the same dataset multiple times; and
  - accuracy (whether full texts were accurate and the version of record; accurate but not the version of record; or inaccurate).
3. Investigation of the available full texts that were missed by EndNote or Zotero in terms of:
  - the reference type;
  - whether texts were available open access or via university subscription; and
  - the content provider or publisher (e.g., Wiley, Science Direct, etc).

Throughout this paper, the term ‘full text’ is used to refer to any item that is available online as an electronic document such as a portable document format (PDF). For this reason, items such as conference abstracts that are available as a PDF are considered full texts for the purposes of this study. The term ‘version of record’ is used to refer to the publisher's final version [10]. Full texts that are not the version of record could contain differences in layout, copyediting, typesetting, and proofreading.

### Understanding the Find Full Text Features

Throughout August and September 2023, e-mail enquiries were made with Clarivate's technical support team and with Zotero's support forum. The enquiries asked which metadata (or lack of metadata) aids or hinders successful retrieval of full texts. Additional contact was made with Clarivate in October 2023 to query whether EndNote 21 (released 19 September 2023) had enhanced performance in finding full texts compared with EndNote 20 [11]. EndNote's webpages on optimizing results using the find full text feature and its frequently-asked-questions page on full texts and PDFs were also used as sources of information [12, 13].

EndNote can search for a maximum of 250 full texts in one go and attach these to the record in the EndNote library. Items searched for are categorized as either: ‘Found PDF,’ ‘Found URL’ (Uniform Resource Locator), or ‘Not Found’.

By default, EndNote can retrieve full text attachments from the Web of Science platform's full text links as well as from PubMed LinkOut [14]. Only the free journal set on the Web of Science platform is checked for all users, whereas users with a subscription may have full IP-based access to all its resources [15]. The digital object identifier (DOI) can help EndNote to retrieve full texts though full texts can still be found without a DOI [16].

If a user has access to an institution's subscriptions, the Open URL and Authentication URL allow some subscription content to be retrieved as a full text and attached to the record. This is set up in the ‘Edit’ menu on EndNote by going to ‘Preferences’ and then ‘Find Full Text’. Institutions using Ex Libris Alma-Primo can enter the details of their link resolver on the same page [17].

The find full text feature is incompatible with content providers that do not allow third-party software to access and retrieve data from them. This applies to open access and subscription content. EndNote's page on optimizing the find full text results lists its incompatibility with: EBSCO; JSTOR; OpenAthens; Wiley; and ScienceDirect [18].

EndNote marks some items as ‘Found URL’ if it cannot find a full text but can find the URL, helping users to access the item or obtain a full text manually (if applicable) [19]. At the time of writing, since the release of version 20.4.1 there have been minor changes to the find full text functionality for EndNote version 20.6, including enhanced full text functions for certain journals and content providers [20]. This information on EndNote's find full text features applies to both EndNote 20 and EndNote 21. Clarivate did not comment on whether EndNote 21 (released 19 September 2023) would have enhanced ability to find full texts over EndNote 20 but described it as having had minimal changes to its full text retrieval features [21].

Zotero, which is open-source software, can look for an unlimited number of full texts in bulk. It can be used for free with no limits on storage space but no cloud storage. For users who require their data to be synced with Zotero's cloud storage (for working collaboratively, or across multiple devices), the program can be used for free with a limit of up to 300 MB data; it also has subscription tiers which determine the storage space per user or institution [22]. Synced libraries can be accessed from the Zotero website without having the software installed [23].

When looking for full texts, Zotero's process is to categorize these as ‘Full Text,’ ‘Accepted Version,’ ‘Submitted Version,’ ‘No PDF Found’ or ‘Failed’. Once found, full texts are attached to the record in Zotero. Zotero also allows subscription content to be retrieved as a full text. Authentication for an institution's subscriptions is set up in the ‘Edit’ menu on Zotero by going to ‘Preferences’ and then the ‘Advanced’ tab. Under ‘Open URL,’ numerous institutions can be selected in the drop-down menu. Alternatively, users can select ‘Custom’ and then paste the OpenURL resolver for their institution.

Zotero uses the DOI or International Standard Book Number to find full texts but can also find full texts without this metadata [24]. The program also uses the metadata for articles on CrossRef, which is used by organizations to register their research and ensure metadata is detailed and accurate [25]. At the time of writing, since the release of version 6.0.27 there have been no changes to the find full text functionality on Zotero affecting the current version 6.0.30 (see: https://www.zotero.org/support/changelog) [26].

### Testing the Performance of EndNote versus Zotero

Four datasets of 250 records (1,000 in total), were taken from three evidence synthesis projects conducted by CRD at the University of York (UoY) and one systematic review by the Cochrane Common Mental Disorders group [27,28, 29, 30].

Two-hundred and fifty records were randomly selected from each dataset using EndNote (due to its use for reference management in the evidence synthesis projects). Only 250 records were used per dataset as this is the maximum number of full texts that EndNote can search for in one go.

Four different healthcare topics were chosen to allow for differences in the full text retrieval due to variations in UoY's subscriptions. There was also variation by reference type: for dataset 1, all records were arranged by reference type in EndNote, and 250 were selected from items marked as ‘journal articles’ in the original library, as this reference type is commonly required by researchers. Datasets 2, 3 and 4 contained mixed reference types to test performance using representative results from evidence synthesis projects. All datasets (sets 1–4) are described below. Although the reference types listed by EndNote will not always be accurate, this was a useful method to provide variety without individually checking each record.

Once each set of 250 records had been selected, they were exported as a .ris file before the find full text process was run separately on EndNote 20 (version 20.4.1) and Zotero 6 (version 6.0.27), using a free account. Each program contained the library authentication details of UoY and were tested individually on the same day and under the same conditions, connected to the University's Virtual Private Network (VPN).

After full texts had been retrieved using both programs, all articles were put into EndNote libraries for each dataset and labeled as either ‘found’ or ‘not found’ and with either ‘EndNote’ or ‘Zotero’ using the ‘custom 4’ field (one of numerous fields on EndNote which can be used for custom annotation of records). Where full texts were found, we investigated whether the attached full text was accurate or inaccurate, and if there were differences in the versions found by each program. A full text attachment was considered accurate if it matched the details in the record, though exceptions were made for minor differences in the publication year, volume, issue, and pagination to allow for variations in the metadata for online, ahead-of-print and printed articles, as well as for metadata errors and updates to publications since retrieval from the databases (dates of the searches are listed below). We created additional categorization for full texts that were accurate but not the version of record, with these items checked by both authors. Items were considered ‘inaccurate’ if the full text was wrong or could not be opened. Accuracy data was labeled in the ‘custom 2’ field. The number of available full texts was determined by adding together the number of full texts available either via EndNote, Zotero, open access or via UoY's access.

To check if a consistent number of full texts was found using the same dataset multiple times, EndNote and Zotero were tested individually on the same day and under the same conditions, whilst connected to the University's VPN. This was only performed for dataset 4, which was checked four times. The records were re-imported each time.

For the various tests of the performance of EndNote versus Zotero, the mean of all four datasets was calculated by adding together all the numbers retrieved from all four datasets and then divided by four. Where necessary, all percentages (or numbers listed as the mean) were rounded up or down to whole numbers.

Details of the datasets are as follows:

1. **Project Title:** Bereavement support and prolonged grief disorder: scoping and mapping the evidence.
  **Databases Searched:** 28 October 2022.
  **Reference Types**: 250 Journal Articles.
2. **Project Title:** Do routine surveillance investigations improve survival after paediatric leukaemia? A systematic review.
  **Databases Searched:** 5–7 December 2022.
  **Reference Types**: 221 Journal Articles, 1 Book, 8 Reports, 20 Web Pages.
3. **Project Title:** Communicating cardiovascular risk: Systematic review of qualitative evidence.
  **Databases Searched:** 8 November 2022.
  **Reference Types**: 239 Journal Articles, 1 Thesis, 8 Books, 2 Book Sections.
4. **Project Title:** Digital mental health interventions for treating depression in adults in low- and middle-income countries.
  **Databases Searched:** 27–29 March 2023.
  **Reference Types**: 186 Journal Articles, 48 Theses, 16 Web Pages.

**Figure 1:**
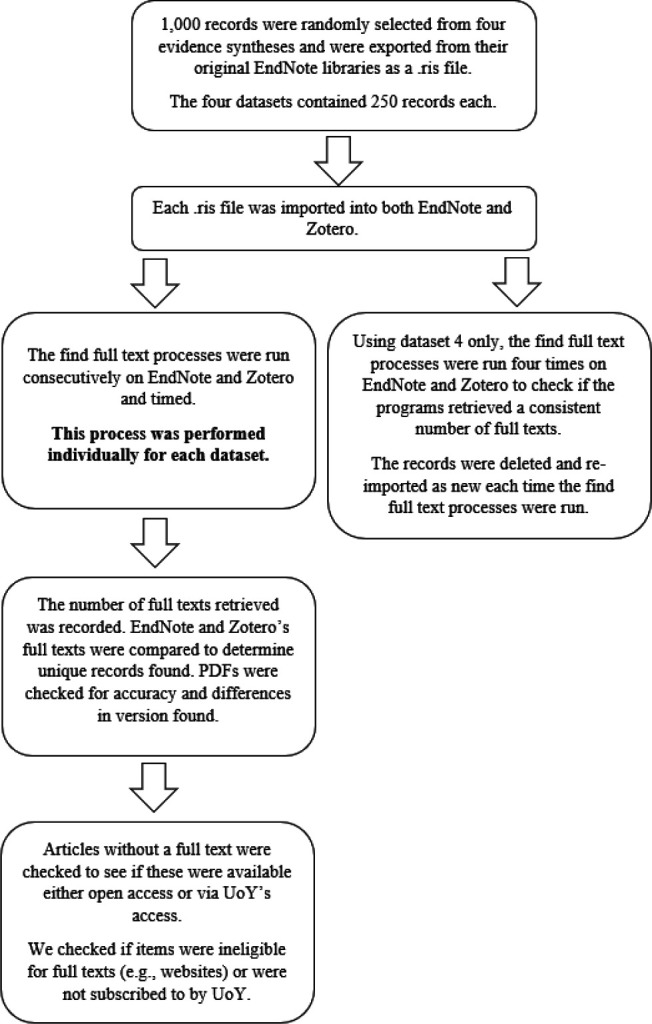
Testing the Performance of EndNote versus Zotero

The process of testing the performance of EndNote versus Zotero is summarized in Figure 1.

### Full Texts Missed by EndNote or Zotero

Any full texts that were missed by EndNote or Zotero were investigated and categorized using annotations in the ‘custom 2’ field in the EndNote libraries used for each dataset.

The following categorizations for articles that were not found by EndNote or Zotero were used:

- not applicable (i.e., any record that is ineligible for a full text attachment such as websites; trial registry records; etc);
- no access (for articles that UoY does not subscribe to);
- insufficient metadata (where there was insufficient metadata in the record to retrieve the article);
- available open access; and
- available via UoY subscription.

The ‘custom 2’ field in EndNote was used to add annotations on the provider of open access and subscription content. In the process of determining open access from subscription access, all items marked as UoY subscription were double-checked using incognito mode on Google Chrome to prevent single-sign-on authentication.

Although not all the categories above are reported on in this paper, full data is included in the supplementary material.

## RESULTS

### Testing the Performance of EndNote versus Zotero:

For each dataset and for each program, Table 1 shows the number of full texts retrieved with either EndNote or Zotero; the number of available full texts (i.e., through EndNote, Zotero, open access or via UoY's access); the percentage of available full texts retrieved; the number of unique full texts retrieved (i.e., found by one program only); the number of different versions of full texts found (where found by both programs); time taken to retrieve the full texts; and means for all columns.

**Table 1:** Retrieval and Time Taken to Retrieve Full Texts

Dataset	Program	FT Retrieval (% of 250)	Available FTs (%of250)	Available FTs Retrieved	Unique FTs	Difference in FT version (of 250)	Time Taken (minutes, seconds)
1	EndNote	91 (36%)	186 (74%)	49%	13	12	16:37
	Zotero	99 (40%)		53%	5		14:40
2	EndNote	57 (23%)	163 (65%)	35%	9	2	18:29
	Zotero	84 (34%)		52%	36		16:45
3	EndNote	103 (41%)	191 (76%)	54%	13	2	21:08
	Zotero	104 (42%)		54%	14		16:22
4	EndNote	92 (37%)	187 (75%)	49%	10	0	15:11
	Zotero	92 (37%)		49%	10		14:38
**Mean**	**EndNote**	**86 (34%)**	**182 (73%)**	**47%**	**11**	**4**	**17:51**
	**Zotero**	**95 (38%)**		**52%**	**16**		**15:36**

**FT** = Full Text

The mean number of full texts retrieved was 86 (34%) for EndNote versus 95 (38%) for Zotero, as numerous records from each dataset were not applicable (i.e., clinical trial records, websites, etc) or not accessible via UoY's subscriptions. However, EndNote retrieved 47% of available full texts versus 52% for Zotero.

Both EndNote and Zotero found unique full texts. The mean number of unique full texts identified by EndNote was 11 versus 16 for Zotero. Figures were relatively consistent between datasets, except for dataset 2 which had nine unique full texts found by EndNote versus 36 by Zotero.

Three out of four datasets contained different full text versions found by EndNote and Zotero for the same record. For datasets 2 and 3, only two records had different full text versions, compared with 12 different versions in dataset 1. The mean number of differences in the full text version per dataset was four.

The time taken to retrieve the full texts on both EndNote and Zotero was not vastly different between datasets. It took EndNote a mean of 17 minutes and 51 seconds per dataset, versus a mean of 15 minutes and 36 seconds for Zotero. The biggest difference in time taken for a single dataset was for dataset 3, which took EndNote 21 minutes and 8 seconds, compared to 16 minutes and 22 seconds by Zotero [31].

For each dataset and for each program, Table 2 shows the total number of accurate full texts retrieved, which are then broken down into the number of accurate full texts which were or were not the version of record. The table also shows the total number of inaccurate full texts, and the reason full texts were considered inaccurate. Columns which report on the number of full texts that were or were not the version of record show percentages out of the total number of accurate full texts.

**Table 2:** Retrieval and Time Taken to Retrieve Full Texts

Dataset	Program	Accuracy			Inaccuracy	
		Total Accurate	Accurate: version of record	Accurate: not version of record	Total Inaccurate	Inaccurate Reason
1	EndNote (n = 91)	90 (99%)	81 (90%)	9 (10%)	1 (1%)	1 wrong article
	Z otero (n = 99)	98 (99%)	92 (94%)	6 (6%)	1 (1%)	1 wrong article
2	EndNote (n = 57)	57 (100%)	56 (98%)	1 (2%)	0 (0%)	
	Z otero (n = 84)	84 (100%)	84 (100%)	0 (0%)	0 (0%)	
3	EndNote (n = 103)	102 (99%)	83 (81%)	19 (19%)	1 (1%)	1 wrong article
	Z otero (n = 104)	103 (99%)	83 (81%)	20 (19%)	1 (1%)	1 wrong article
4	EndNote (n = 92)	90 (98%)	89 (99%)	1 (1%)	2 (2%)	1 wrong article 1 corrupt file
	Z otero (n = 92)	90 (98%)	87 (97%)	3 (3%)	2 (2%)	1 wrong article 1 corrupt file
**Mean**	**EndNote**	**85 (99%)**	**77 (91%)**	**8 (9%)**	**1 (1%)**	
	**Zotero**	**94 (99%)**	**87 (93%)**	**7 (7%)**	**1 (1%)**	

Although EndNote found fewer accurate full texts compared to Zotero (a mean of 85 versus 94, respectively), for both programs, 99% of the retrieved full texts were accurate. The mean number of accurate full texts that were not the version of record was eight (9%) for EndNote, compared to seven (7%) for Zotero.

Table 3 reports on the number of full texts retrieved by EndNote and Zotero (out of 250 records taken from dataset 4 only) when the dataset was newly-imported into each program. The date the find full text processes were run is included in Table 3.

**Table 3:** Consistency of EndNote and Zotero

Date Run		29/08/2023	21/09/2023			
Dataset	Program	Original FT Retrieval	FT Retrieval: 1	FT Retrieval: 2	FT Retrieval: 3	FT Retrieval: 4
4	EndNote	92	90	91	90	91
	Zotero	92	133	92	91	91

**FT** = Full Text

The number of full texts retrieved varied for both programs. Whereas EndNote tended to retrieve a similar number of full texts each time, Zotero was much more variable in the number of full texts retrieved.

Available Full Texts that were Missed

For all datasets, Table 4 shows information about the available full texts that were missed by EndNote or Zotero in terms of the reference type and whether texts were available open access or via UoY's subscriptions.

**Table 4:** Available Full Texts that were Missed

Dataset	Program	Available FTs Missed	Available Reference Types Missed	Open Access Missed	UoY Subscription Missed
1	EndNote	95 (51%)	95 Journal Articles	22 (23%)	73 (77%)
	Zotero	87 (47%)	87 Journal Articles	19 (22%)	68 (78%)
2	EndNote	106 (65%)	104 Journal Articles 2 Reports	84 (79%)	22 (21%)
	Zotero	79 (48%)	77 Journal Articles 2 Reports	60 (76%)	19 (24%)
3	EndNote	88 (46%)	86 Journal Articles 1 Book 1 Thesis	33 (38%)	55 (63%)
	Zotero	87 (46%)	85 Journal Articles 1 Book 1 Thesis	27 (31%)	60 (69%)
4	EndNote	95 (51%)	54 Journal Articles 41 Thesis	56 (59%)	39 (41%)
	Zotero	95 (51%)	54 Journal Articles 41 Thesis	57 (60%)	38 (40%)
**Mean**	**EndNote**	**96 (53%)**	**85 Journal Articles** **0 Books** **1 Report** **11 Thesis**	**49 (51%)**	**47 (49%)**
	**Zotero**	**87 (48%)**	**76 Journal Articles** **0 Books** **1 Report** **11 Thesis**	**41 (47%)**	**46 (53%)**

**FT** = Full Text

For EndNote, 51% of missed full texts were open access and 49% via UoY's subscriptions. Although Zotero's retrieval of full texts was higher, 47% of missed full texts were open access versus 53% available via UoY's subscriptions.

A variety of reference types were missed by EndNote and Zotero. For both programs, the most common missed reference type was journal articles (although this reference type was the most common for each dataset - see the methods section for full details of the reference types included). Other missed reference types were identical in terms of numbers missed by EndNote and Zotero.

Table 5 shows information about the available full texts that were missed by EndNote or Zotero across datasets 1–4 by the 10 most frequent content providers (i.e., publishers, publisher subsidiaries, or platforms hosting published content). The total number of available full texts that were missed by each program is also listed. See the supplementary material for further information on the content providers of available full texts that were missed.

**Table 5:** Available Full Texts that were Missed

EndNote		Zotero	
Provider	Available FTs Missed (N=384)	Provider	Available FTs Missed (N=348)
Science Direct	147	Science Direct	131
Wiley	58	Wiley	39
Sage	38	Sage	33
Taylor & Francis	28	Taylor & Francis	26
Wolters Kluwer	14	Wolters Kluwer	10
ProQuest	10	ProQuest	10
Haematologica	6	Oxford Academic	7
Uppsala Universitet	3	Springer	7
ETHOS	3	ETHOS	3
MAG Online Library	3	MAG Online Library	3

**FT** = Full Text

There is overlap in the most common providers of missed content. For both EndNote and Zotero, the top six providers were Science Direct, Wiley, Sage, Taylor&Francis, Wolters Kluwer, and ProQuest.

## DISCUSSION

### Understanding and Using EndNote and Zotero's Find Full Text Features

There are several differences between EndNote and Zotero's find full text features worth commenting on. Firstly, EndNote can only look for a maximum of 250 full texts in one go. This means larger datasets may have additional time-savings when run on Zotero since this program was faster and is not limited in the number of full texts it can search for in one go. Secondly, for items not found as full texts, EndNote can find the URL and update the record, helping users find full texts manually. In comparison, Zotero can access additional metadata via CrossRef but does not attach this to the record or correct any differences in the metadata. Thirdly, Zotero's process of categorizing items sought as full text as either ‘Full Text,’ ‘Accepted Version,’ ‘Submitted Version,’ ‘Not Found,’ or ‘Failed’ is more transparent in alerting users to the full text versions retrieved.

Once full texts have been found, these can be read (and annotated) inside either EndNote or Zotero or accessed outside the programs using a PDF reader. PDFs or other document formats can be individually attached to the record in either program. Both programs allow multiple attachments per record, which is helpful for users who may want to screen supplementary material alongside the full text paper.

For researchers screening using dedicated systematic review software, Zotero is unique in being able to interface with EPPI-Reviewer to bulk import full texts and automatically attach these to the record in EPPI-Reviewer [32]. As use with EPPI-Reviewer requires syncing data to cloud storage, the available storage space will vary with the type of subscription to Zotero. In comparison, Covidence and Rayyan cannot interface with either EndNote or Zotero. However, Covidence and Rayyan allow bulk-import of PDFs which then automatically attach to a record [33, 34, 35]. For use with Covidence, Rayyan, or other software allowing bulk-imports, PDFs could have been found and copied from EndNote and/or Zotero or found manually.

### Testing the Performance of EndNote Versus Zotero

It is important for users to check whether full texts are the version of record. Notably, all datasets contained full texts that were not the version of record. This was the case for 19% of the full texts retrieved by EndNote and Zotero for dataset 3.

Investigating the unique full texts retrieved by EndNote or Zotero led to the finding that some content providers restrict access to open access content. As an example, a PDF hosted by publisher Mary Ann Liebert [36] denied UoY access even though it was available open access on PubMed Central [37]. Only Zotero was able to retrieve this full text. Double-checking items to ensure they are not available elsewhere could save money for orders placed for full texts (e.g., from copyright libraries such as the British Library, through the purchase of online articles from publisher websites, etc).

EndNote and Zotero tended to make the same mistakes for the few full texts that were inaccurate, though there were minor differences in their inaccuracies. For datasets 1 and 3, the two incorrect full texts were for the same record and had the same incorrect attachment. Similarly, the one corrupt file found for dataset 4 was for the same record. For dataset 3, EndNote and Zotero each found one incorrect full text but for different records.

Additional testing of retrieval comparing EndNote 20 (20.4.1) with EndNote 21 (version 21.2) for all datasets is not reported on in this paper but is available in the supplementary material.

### Full Texts Missed by EndNote or Zotero

Full texts could have been missed for multiple reasons. As noted previously, some content providers prevent third-party software retrieving full texts. Another cause could be suboptimal metadata (either in the original database export or when records were first imported into EndNote). Although this study does not report on the database of origin for the records tested, in some cases the metadata exported from the databases could play a role in whether a full text is found. As an example, the database Social Care Online (SCIE) uses the article's record on the database as the URL rather than linking the user to the article on the publisher's website; it also does not always include the DOI. This could lead to EndNote or Zotero missing full texts. Users could check the metadata in their database exports to ensure DOIs and URLs are being imported. On numerous occasions, we found that a full text PDF was not retrieved by EndNote or Zotero even though the URL linked directly to it. It is unclear to what extent the inclusion or absence of DOIs or URLs affects full text retrieval, as Zotero has access to additional metadata via the use of CrossRef. The authors were unable to investigate this as they are not software developers.

The supplementary material was cross-referenced to analyze data by reference types. This revealed that ‘journal articles’ were the only reference type found as full texts by either EndNote or Zotero, though the datasets only included small numbers of other reference types. However, as the second most common reference type, none of the 48 theses were retrieved by EndNote or Zotero, even though 41 were available. This finding may not be generalizable but could be useful information for those searching thesis repositories.

There was overlap in the most common providers of missed content between EndNote and Zotero. Clarivate lists some of these content providers as being incompatible with EndNote [38], and Zotero is also likely to be incompatible with certain providers for the same reasons. Table 5 may highlight where one program is more likely to retrieve full texts from some of these providers over the other. However, this is not necessarily the case if, for example, alternative copies of these full texts were retrieved from a different provider. For instance, the earlier example of the Mary Ann Liebert article available open access on PubMed Central [39] found only by Zotero, may suggest Zotero can access PubMed Central. But as Zotero uses additional metadata from CrossRef, we cannot be certain from which provider a full text was retrieved without further investigation. This means there are too many variables to take Table 5 at face value, even with cross-referencing of the additional information in the supplementary material.

This is an exploratory study based on software and data that was readily available to the authors. Only 1,000 records taken from healthcare literature were tested using UoY's subscriptions in August and September 2023. EndNote 20 (version 20.4.1) and Zotero 6 (version 6.0.27) were used and there have been further enhancements to EndNote's find full text functions for certain journals and content providers since the tests performed in this paper [40].

As all 1,000 records were randomly selected using EndNote (due to its use for reference management in the evidence syntheses projects) all records were subject to EndNote's import filters for each database. It is possible that there could be subtle variations in metadata if database exports were imported directly into both EndNote and Zotero. Moreover, records from some of the databases used in the evidence syntheses were imported using adapted or custom import filters for EndNote.

Reference types of articles used in the datasets were determined by how these were automatically categorized in the original EndNote libraries of the evidence syntheses and were not checked.

Only one content provider was annotated for each missed full text available open access or via UoY's subscriptions, even though some were available from multiple providers.

The time taken for EndNote and Zotero to find full texts may have been affected by computer performance and internet connection.

EndNote and Zotero do not find all available full texts. Users should not assume full text attachments are correct, are the publisher's final version, or that records without attachments cannot be retrieved manually. It is possible that repeating the full text retrieval process multiple times could yield additional full texts. Since both programs found unique full texts, where EndNote has been used for full text retrieval, it may be useful to look for any remaining items without a full text using Zotero (or vice versa), if users have access to both programs.

The performance of EndNote and Zotero was similar in many respects with one exception: Zotero was much more variable in the number of full texts retrieved when testing the same dataset for full text retrieval multiple times. However, for every dataset, Zotero found equal to or more than the number of full texts found by EndNote.

Zotero was superior in terms of the number of full texts retrieved (finding 52% of those available versus 47% by EndNote) and in finding the version of record (at 93% versus 91% by EndNote). Zotero was also more transparent in terms of which version of a full text was found and was faster than EndNote by a mean of 2 minutes and 15 seconds. For both programs, 99% of the retrieved full texts were accurate.

Overall, the findings are informative for information specialists, researchers, and institutions who may want to decide whether to use one program over the other or both together.

## Data Availability

Data associated with this article are available in the supplementary material.
